# Impaired bone matrix maturation and mineralization are prevalent in adolescent end-stage kidney disease

**DOI:** 10.1093/jbmrpl/ziag036

**Published:** 2026-03-12

**Authors:** Nadja Fratzl-Zelman, Renata C Pereira, Stéphane Blouin, Markus A Hartmann, Kathleen Noche, Isidro B Salusky, Katherine Wesseling-Perry

**Affiliations:** 1st Medical Department Hanusch Hospital, Ludwig Boltzmann Institute of Osteology at the Hanusch Hospital of OEGK and AUVA Trauma Centre Meidling, Vienna, Austria; Vienna Bone and Growth Center, Vienna, Austria; Department of Pediatrics, David Geffen School of Medicine at UCLA, Los Angeles, CA 90095, United States; 1st Medical Department Hanusch Hospital, Ludwig Boltzmann Institute of Osteology at the Hanusch Hospital of OEGK and AUVA Trauma Centre Meidling, Vienna, Austria; Vienna Bone and Growth Center, Vienna, Austria; 1st Medical Department Hanusch Hospital, Ludwig Boltzmann Institute of Osteology at the Hanusch Hospital of OEGK and AUVA Trauma Centre Meidling, Vienna, Austria; Vienna Bone and Growth Center, Vienna, Austria; Department of Pediatrics, David Geffen School of Medicine at UCLA, Los Angeles, CA 90095, United States; Department of Pediatrics, David Geffen School of Medicine at UCLA, Los Angeles, CA 90095, United States; Department of Pediatrics, David Geffen School of Medicine at UCLA, Los Angeles, CA 90095, United States; Phoenix Children’s Hospital, University of Arizona College of Medicine, Phoenix, AZ 85016, United States

**Keywords:** bone mineralization, osteocytes, FGF23, histomorphometry, quantitative backscattered electron imaging (qBEI)

## Abstract

Children and adolescents with end-stage kidney disease (ESKD) have a high prevalence of bone deformities and fractures, even when bone histomorphometric measures of bone formation, mineralization, and volume are within normal range. We postulated that bone matrix quality and osteocyte differentiation are impaired in ESKD in ways which might be not reflected by alterations in surface-based cell activity. We assessed bone turnover, mineralization, and volume by traditional histomorphometry in bone biopsy cores from 24 adolescent patients with ESKD. We stained sequential sections of the same cores for apoptosis (TUNEL staining) and for osteocyte-specific maturation markers (sclerostin, DMP1, and FGF23). We then performed quantitative backscattered electron imaging (qBEI) to assess bone mineralization density distribution on the whole bone surface and additionally in bone packets containing FGF23-expressing osteocytes. Increased amounts of osteoid (OV/BV > 4.7%) and a true defect in bone surface mineralization (O.Th > 10 μm combined with MLT > 27.1 d) were detectable by histomorphometry in 29% and 17% of bone samples, respectively. By contrast, qBEI detected an excess in primary mineralizing bone (TbCaLow > 6.8%) in 88% of samples and abnormally low bone matrix mineralization (TbCaMean < 22.1 wt% calcium) in 75% of samples. Osteocyte lacunar density and size were within normal range. Unexpectedly, higher numbers of FGF23-expressing osteocytes and a more elongated osteocyte lacunar shape were found in bone samples with greater matrix mineralization. In conclusion, matrix mineralization defects, which are not detected by traditional histomorphometry, are highly prevalent in ESKD bone and reflect poor bone matrix maturation. High numbers of FGF23-expressing osteocytes are found in bone with the most appropriate matrix maturation characteristics. This differs from the bone characteristics of individuals with normal kidney function, in whom large amounts of FGF23 result in low circulating phosphate levels, which in turn triggers severe osteomalacia and hypomineralized periosteocytic lesions.

## Introduction

Bone deformities, fractures, and impaired growth are common features of childhood CKD that contribute to significant lifetime morbidity.^1^ The Turnover, Mineralization, Volume (TMV) classification system for CKD-mediated bone disease was established in 2006 and is still the gold standard for the diagnosis of renal osteodystrophy (ROD).^2^^,^^3^ This classification system is based on results from bone histomorphometric analysis of iliac crest biopsy specimens and is limited to assessing cell activity on the surface of bone. Using this TMV system, an excess of unmineralized bone matrix (osteoid) and slowed mineralization rates are observed by traditional histomorphometry on bone biopsy samples in all CKD stages.^4^^,^^5^ However, neither the prevalence of hyperparathyroidism nor of surface mineralization defects fully accounts for the prevalence of bone fragility in the CKD population.^4^^,^^6^ Bone strength and bone material properties are heavily influenced by the degree of mineralization of the bone matrix, which is controlled at the cellular level by osteocyte maturation within the collagenous matrix.^7^ Matrix mineralization cannot be measured by traditional histomorphometry but is assessed by additional techniques, such as quantitative backscattered electron microscopic imaging (qBEI). Previous data suggest that, in children and adolescents with CKD, bone matrix mineralization may not always correlate with the mineralization defects that are present on the bone surface.^8^ However, the quality of bone matrix in CKD bone, and how it relates to bone surface histomorphometry, has not been fully characterized.

We have previously reported a generalized impairment in osteoblast and osteocyte maturation in the uremic milieu.^9^ Fibroblast growth factor 23 (FGF23)-expressing osteocytes co-express the very early differentiation marker e11/gp38.^10^ These osteocytes are embedded in bone packets that are in an early stage of secondary mineralization and are thus not yet fully mineralized.^10^ Increased numbers of FGF23-expressing osteocytes are found in the bone of many CKD patients,^9^ and we have demonstrated an inverse relationship between numbers of these FGF23-expressing osteocytes and the accumulation of unmineralized osteoid that is laid down by osteoblasts and that is quantifiable by bone histomorphometry.^9^^,^^10^ This suggests a crosstalk between young, FGF23-expressing, osteocytes and the maturation of osteoblasts on the bone surface. A similar interaction between osteocytes and surrounding bone matrix quality is also likely, although this has never been studied. We thus postulated that local matrix mineralization defects might be prevalent in CKD bone, that these defects might not be fully captured by traditional histomorphometry, and that defective matrix mineralization might be related to impairments in osteocyte-specific protein expression and maturation.

In order to better understand the relationships between cellular activity at the bone surface, osteocyte maturation, and the associated mineralization of the bone matrix in young patients with advanced (end-stage) CKD (ESKD), we combined previously obtained biochemical and bone histomorphometric data with concurrently obtained immuno-histomorphometric assessment of osteocyte-specific protein expression and qBEI. This integrative approach enabled a comprehensive analysis of the relationships among circulating markers of mineral balance, bone surface remodeling activity, matrix mineralization quality, osteocyte lacunar structure, and indicators of osteocyte maturation. By combining traditional bone histomorphometry with immunostaining and high-resolution imaging of the mineralized matrix, we were able to examine both cellular and structural abnormalities that associate with bone disease and impaired mineralization in CKD.

## Materials and methods

### Overall study design

The overall design of the study is shown in Figure S1.

### Bone biopsy samples

Transiliac bone biopsy specimens from 24 patients with ESKD, ages 8-25 yr (median 17.1 (IQR: 15.1, 19.9) yr), were included in this analysis. Samples were selected based on the presence of high turnover or low-to-normal turnover ROD. In the high turnover cohort, BFR/BS was *>*63 μm^3^/μm^2^/yr (*n* = 10). In those with low-to-normal turnover, BFR/BS was *<*32 μm^3^/μm^2^/yr (*n* = 14). Reference values for healthy children and adolescents were from Glorieux et al.^11^ All biopsy samples were previously performed as part of clinical studies.^4^^,^^12^ Informed consent was obtained for these studies as detailed in previous manuscripts^4^^,^^12^ and approval was obtained from the UCLA IRB for the current analyses. Demographic data, biochemical values, and traditional bone histomorphometric values from these samples were previously described and are shown in Table S1.^4^^,^^12^

### Characterization of bone immunohistochemistry and apoptosis

Immunohistochemistry was performed to assess bone protein expression in undecalcified sections as previously reported.^9^ In brief, 5 μm sections of bone were deplasticized in xylene and chloroform, rehydrated in graded alcohol solutions, and partially decalcified in 1% acetic acid. Endogenous peroxidase activity was quenched in 3% hydrogen peroxide/methanol solution. Nonspecific binding was blocked in avidin-biotin solution and in 5% normal horse serum with 1% bovine serum albumin. Sections were incubated with affinity purified polyclonal goat anti-human FGF23 (225-244) (Quidel), monoclonal mouse anti-human matrix extracellular phosphoglycoprotein (MEPE) (LFMb33) (42-525) (provided by Dr. Larry Fisher, National Institutes of Health), monoclonal mouse anti-human dentin matrix protein 1 (DMP1) (LFMb31) (62-513) (Dr. Larry Fisher), and mouse anti-human sclerostin (R&D Systems) primary antibody overnight at 4 °C in a humidified chamber. Samples were then incubated with biotinylated horse anti-goat or mouse secondary antibody (Vector) followed by ABComplex/HRP complex (Vector) and developed using AEC kit (Vector). Sections were counterstained with Mayer hematoxylin (Sigma-Aldrich).^9^

Apoptosis in nondecalcified bone sections was assessed via in situ TUNEL reaction in the samples using Klenow terminal deoxynucleotidyl transferase per manufacturer’s instructions (Oncogene Research Products). Undecalcified bone sections were deplasticized, permeabilized with 20 μg/mL proteinase K, and endogenous peroxidase activity was quenched using 3% hydrogen peroxide in methanol. Fragmented DNA ends were labeled using Klenow terminal deoxynucleotidyl transferase. Signal detection was achieved with streptavidin-conjugated peroxidase and 3,3′-diaminobenzidine, followed by counterstaining with methyl green. Negative controls included sections incubated with vehicle only, while positive controls were generated by pre-treating sections with DNase I.

Immunoreactivity for FGF23 and MEPE along with TUNEL staining (for osteocyte apoptosis) were quantified by counting the number of nuclei staining positive and normalizing this to bone area. FGF23, along with sclerostin and DMP1, was also quantified using the Ariol scanning system. All slides were scanned at 20x magnification with a red filter and digitized (Applied Imaging Inc.). Analyzed fields were manually selected to avoid areas with tissue damage or artifacts occurring during immunostaining. FGF23, DMP1, and sclerostin were expressed as pixels/mm^2^.^13^

### Characterization of bone matrix mineralization

Matrix mineralization was evaluated by qBEI and data obtained in this study were compared with previously published pediatric reference values (*n* = 50, age-range: 2-20.9 yr).^14^ In preparation for qBEI, the surface of each bone sample was gently ground with a series of carbide grinding papers and polished with 3 μm—followed by 1 μm—of polycrystalline diamond spray (Logitech PM5, Logitech Ltd.). As little bone was removed as possible in order to maintain the proximity of the qBEI surface to the FGF23-stained section. The bone surface was then coated with a thin carbon layer by vacuum evaporation (Agar Scientific Ltd.). Quantitative backscattered electron imaging was performed using a field emission scanning electron microscope (FESEM SUPRA 40, Zeiss) equipped with a 4-quadrant semiconductor backscattered electron detector (20 kV accelerating voltage, 270-320 pA probe current, and 10 mm working distance). Backscattered electron images (1024 × 768 pixels, 8 bits) were captured at a resolution of 1.76 μm/pixel and 0.88 μm/pixel, for bone images and FGF23-expressing bone packets, respectively.

In qBEI, the number of backscattered electrons is linearly dependent on the average local atomic number of the sample for low values of *Z*. Thus, a calibration of the SEM with standards of known *Z* numbers provides a quantitative relationship between the measured gray level (GL) and a local mineral content in bone. During calibration, the brightness and contrast of the detector are tuned in order to obtain a GL of 25 ± 1 for carbon (*Z* = 6) and 225 ± 1 for aluminum (*Z* = 13). After calibration, the measured GL is related to a mineral content as weight % calcium = 0.1733 × GL^−4.332^.^15^ The GL image frequency histogram is converted to a mineral content frequency histogram, termed the bone mineralization density distribution (BMDD). BMDD is described by the following parameters: CaMean (weight % calcium, average calcium concentration of the bone area), CaPeak (weight % calcium, most frequently occurring calcium concentration), CaWidth (Δ weight % calcium, width of the BMDD curve at half maximum), CaLow (% bone area, percentage of bone tissue mineralized at <fifth percentile of healthy reference values), and CaHigh (% bone area, percentage of bone tissue mineralized at >95th percentile of healthy reference values).^15^

BMDD was analyzed separately in total trabecular (Tb) and cortical (Ct) cross-sectional area of each bone sample (resolution 1.76 μm/pixel) as well as in bone packets containing FGF23-expressing osteocytes (resolution 0.88 μm/pixel). All BMDD measures were obtained from the bone surface directly below the section used for FGF23 immunohistochemical staining. Acquired measurements were mapped according to FGF23 staining obtained in the adjacent histological section.^10^ BMDD values from trabecular and cortical bone were compared to published reference ranges obtained from a healthy cohort of children and adolescents (*n* = 50, age-range 2-20.9 yr^14^) and were deemed as “increased” or “decreased” when above or below the IQR.

### Characterization of osteocyte lacunae sections

Osteocyte lacunae sections (OLS) analyses were performed on qBEI images obtained as described above. At least 25 backscattered electron microscopy images of 1024 × 768 pixels were captured from the cortical and trabecular compartments with a pixel size of 0.88 μm. An in-house custom-made macro in ImageJ software (version 1.52; NI) was used to set a mineralization threshold (5.2 wt% calcium) to discriminate osteocyte lacunae from surrounding mineralized bone matrix and a size threshold between 5 and 200 μm^2^ to distinguish the OLS from larger channels, as described previously.^14^ The five parameters describing the OLS (OLS-porosity, OLS-density, OLS-area, OLS-perimeter, and OLS-aspect ratio) are described elsewhere.^16^

### Statistical analysis

Measurements are reported as median (IQR) or mean *+* SE as appropriate. Between group comparisons were performed using the Wilcoxon rank sum or Kruskal–Wallis test. The Spearman correlation coefficient was used to assess the monotonic relationships between variables. Multivariable linear regression was also used to assess the relationship between predictors and outcomes controlling for covariates. Statistical analyses were performed using SAS software (SAS Institute Inc.) or GraphPad Software (version 10.6.1) and all tests were 2-sided. A probability of type I error less than 5% was considered statistically significant and ordinary *p* values are reported.

## Results

### Patients, biochemical values, and classification according the TMV system

Transiliac crest bone biopsy samples were performed at UCLA between September of 2007 and May of 2012. The demographic data and biochemical data from the patients included in this analysis (total ESKD cohort) are listed in Table S1. Samples were obtained from patients treated with peritoneal dialysis (*n* = 17) and from patients treated with hemodialysis (*n* = 7); 17 were from boys and 7 were from girls with ESKD due to glomerulonephritis (GN, *n* = 6), congenital anomalies of the kidney and urinary tract (CAKUT, *n* = 10), or other (*n* = 8). Most (*n* = 16) patients had been diagnosed with ESKD within 2 yr of the bone biopsy; however, there was a significant minority (*n* = 8) who had longer-standing ESKD of 2-20 yr duration. Serum calcium levels were in the normal range, while serum phosphate and plasma intact and C-terminal FGF23 concentrations were elevated in most patients. As assessed by tetracycline labeling on histomorphometry, fourteen patients had low/normal bone formation rates, while 10 had high bone formation rates. Eroded surfaces (ES/BS) and osteoid indices (OS/BS, OV/BV, and O.Th) were higher in the high BFR/BS group, while mineralization lag time (MLT) and osteoid maturation time were increased in those with low/normal bone formation. There were no differences in trabecular bone volume (BV/TV) or mineralized trabecular bone volume (Md.BV/TV, assessed by qBEI) between those with high and those with low/normal rates of bone formation. In addition, differences in serum calcium, phosphorus, intact FGF23, and C-terminal FGF23 were not observed. As expected, serum alkaline phosphatase (ALP) as well as PTH were higher in the high BFR/BS group (all data are compiled in Table 1).

**Table 1:** Demographic, biochemical, and bone histomorphometric values in samples with high (BFR/BS > 63 μm^3^/μm^2^/yr) and low/normal (BFR/BS < 32 μm^3^/μm^2^/yr) bone formation rates.

**Demographics**				
	High bone formation rate (*n* = 10)	Low/normal bone formation rate (*n* = 14)	*p* value	
**Age (yr)**	17.1 (15.0, 18.9)	17.5 (15.7, 21.0)	.396	
**Sex (M/F)**	8/2	9/5	.404	
**Underlying disease (*n*)** **IC-MPGN** **FSGS** **Alport’s disease** **CAKUT** **ADPKD** **Cystinosis** **Cystic NOS** **Unknown**	2 4 2 2	5 1 1 1 4		
**Biochemicals**				
				Normal ranges
**Calcium (mg/dL)**	9.0 (7.9, 9.4)	9.3 (8.6, 9.6)	.401	8.4-10.2
**Phosphorus** **mg/dL** **Z-score**	7.4 (5.7, 8.6) 4.9 (2.2, 6.7)	5.7 (5.0, 6.6) 2.1 (0.8, 3.3)	.203 .226	age specific
**ALP (IU/L)**	256 (154, 384)	95 (79, 152)^a^	.013	Age and lab specific
**PTH (pg/mL)**	923 (574, 1255)	339 (113, 559)^a^	.019	10-65
**Intact FGF23 (pg/mL)**	865 (360, 4707)	2190 (171, 5718)	.594	<100
**C-terminal FGF23 (RU/mL)**	1622 (1147, 5577)	3111 (337, 4642)	.476	<100
**Trabecular bone histomorphometry**				
				Normal range^11^
**Turnover**				
**Bone formation rate** **(BFR/BS; μm** ^**3**^**/μm** ^**2**^**/yr)**	75.6 (67.7, 92.1)	8.8 (0.3, 26.8)^b^	<.001	39.3 ± 17.5 35.5 (6.8-78.4)
**Eroded surface** **(ES/BS; %)**	12.7 (11.0, 15.5)	5.1 (1.6, 11.3)^a^	.018	16.6 ± 5.6 15.6 (8.5-32.5)
**Mineralization**				
**Osteoid surface** **(OS/BS; %)**	32.4 (28.6, 47.5)	17.2 (7.7, 35.8)^a^	.028	24.9 ± 10.0 22.5 (4.9-54.3)
**Osteoid volume** **(OV/BV; %)**	4.6 (3.3, 9.8)	2.1 (0.8, 4.5)^a^	.013	2.4 ± 1.2 2.26 (0.41-4.15)
**Osteoid thickness** **(O.Th; μm)**	11.6 (9.5, 13.8)	7.4 (6.2, 9.5)^b^	.008	6.4 ± 1.4 6.1 (3.9-10)
**Mineralization lag time (MLT; d)**	20.5 (11.6, 27.4)	48.5 (29.5, 109.9)^b^	.003	15.5 ± 4.8 15. 1 (8.7-29.1)
**Osteoid maturation time (OMT; d)**	11.0 (8.3, 14.2)	16.8 (10.7, 22.2)^a^	.016	7.3 ± 1.9 6.9 (4.4-11.9)
**Volume**				
**Bone volume (BV/TV; %)**	37.3 (30.0, 39.9)	30.5 (26.2, 32.5)	.107	23.3 ± 5.3 24.0 (13.5-35.5)
**Mineralized bone volume (Md.BV/TV; %)**	35.7 (29.7, 37.9)	31.0 (25.0, 31.7)	.074	23.3 ± 5.4 23.3 (12.9-35.1)

Data are reported as median with IQR. Reference values are reported as mean ± SE and median (range).

a *p* < .05 between high-normal to high and low-normal to low turnover groups.

b *p* < .01 between high-normal to high and low-normal to low turnover groups.

Abbreviations: ADPKD, autosomal dominant polycystic kidney disease; CAKUT, congenital anomalies of the kidneys and urinary tract; FSGS, focal segmental glomerulosclerosis; IC-MPGN, immune complex membranoproliferative glomerulonephritis; NOS, not otherwise specified.

### Mineralization defects are prevalent in ESKD bone, but histomorphometry and qBEI have different sensitivities for these defects

**Figure 1:**
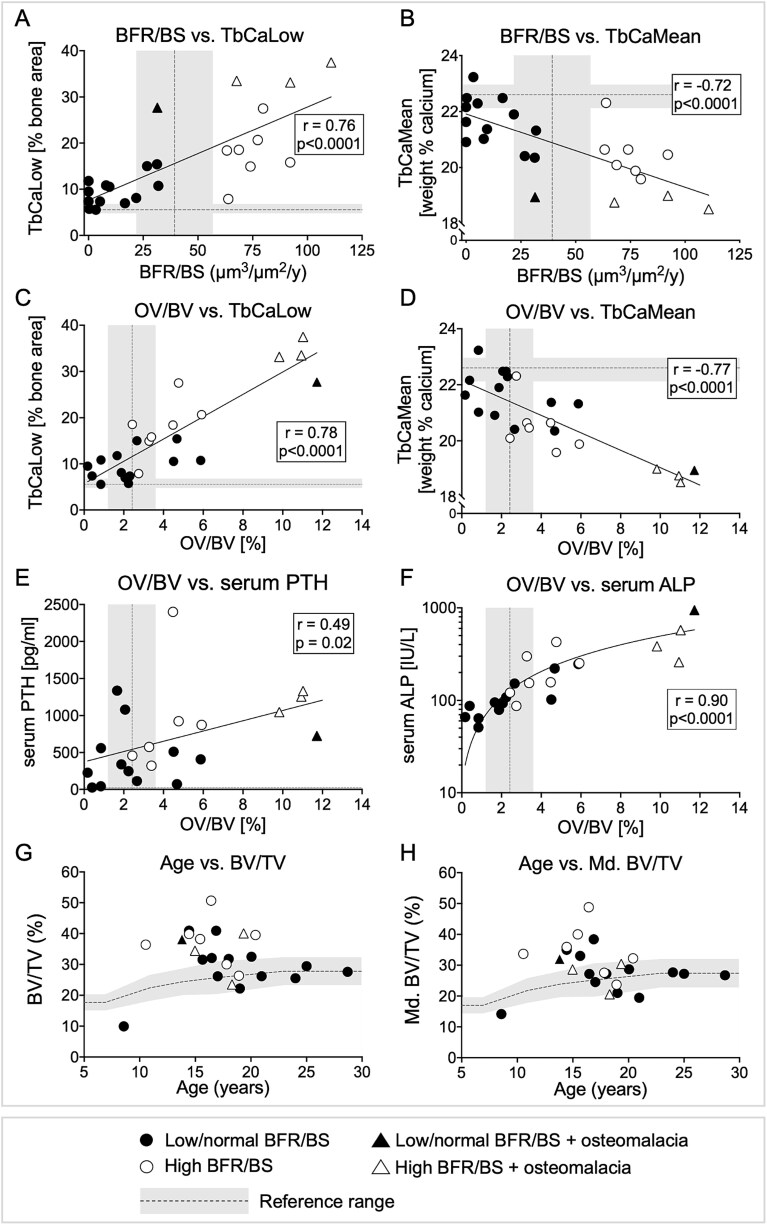
Scatterplots depicting relationships between histomorphometric parameters, qBEI parameters, and biochemical variables. (A) Percentage of lowly mineralized trabecular bone (TbCaLow; qBEI measure) expressed as a function of bone formation rate (BFR/BS; histomorphometric measure). (B) Average calcium in trabecular bone packets (TbCaMean; qBEI) expressed a function of bone formation rate (histomorphometry). (C) Percentage of lowly mineralized trabecular bone (TbCaLow; qBEI) expressed as a function of osteoid volume (OV/BV; histomorphometry). (D) Average calcium in trabecular bone packets (TbCaMean; qBEI) expressed as a function of osteoid volume (OV/BV; histomorphometry). (E) Serum PTH concentration as a function of osteoid volume (OV/BV; histomorphometry). (F) Serum ALP expressed as a function of osteoid volume (OV/BV; histomorphometry). (G) Bone volume (BV/TV; histomorphometry) expressed as a function of age in years. (H) Mineralized bone volume (Md.BV/TV; qBEI) expressed as a function of age in years. Normal ranges are demarcated by the shaded areas. Open symbols depict patients with high bone turnover; closed symbols depict patients with low/normal bone turnover; triangles depict patients meeting histomorphometric criteria for defective mineralization (osteomalacia). A linear regression line was used to fit the whole dataset in each graph; the r value of the line and the *p*-value of the correlation are displayed in the graph. Reference ranges (gray bands, with the line in the center indicating the IQR and the median value) for BMDD parameters are from Mähr et al.,^14^ for bone histomorphometry parameters from Glorieux et al.,^11^ and for FGF23/bone area from Pereira et al.^9^

A summary of qBEI parameters of the total ESKD cohort are displayed in Table S2. In general, trabecular bone was under-mineralized while cortical bone mineralization was better preserved compared to reference ranges. Marked differences in the degree of bone matrix mineralization were observed between the high and the low/normal bone formation groups. The average (CaMean) and the most frequent (CaPeak) calcium content of all samples were lower than reference values. However, TbCaMean was higher in samples with low/normal than in samples with high BFR/BS. In cortical (Ct) bone, CaMean was modestly decreased in bone with high (compared to bone with low/normal) BFR/BS. In both trabecular and cortical bone, the heterogeneity in mineralization (CaWidth) and the percentage of lowly mineralized bone matrix (CaLow) were increased in samples with high bone formation rates as compared to those with low/normal bone formation rates. No between-group differences were observed in the percentage of highly mineralized trabecular bone matrix (TbCaHigh), while CtCaHigh was increased in samples with low/normal as compared to those with high BFR/BS (Table 2). All cortical BMDD parameters were within reference ranges in samples with low/normal BFR/BS. With the exception of TbCaHigh in samples with low/normal BFR/BS, all trabecular bone BMDD parameters, in both groups, fell outside reference ranges (data not shown). Consistent with studies in other populations^17^^,^^18^ and consistent with the physical process of new bone mineral accretion, histomorphometric measures of bone formation (BFR/BS), and osteoid deposition correlated directly with TbCaLow and inversely with TbCaMean (Figure 1A-D). Similarly, serum PTH and serum ALP levels correlated directly with TbCaLow and OV/BV and inversely with TbCaMean (Table 3, Figure 1E and F).

**Table 2:** Bone matrix material properties by quantitative backscattered electron imaging (qBEI) and bone protein expression by immunohistochemistry in samples with high and low/normal bone turnover.

	**High bone formation rate (*n* = 10)**	**Low/normal bone formation rate (*n* = 14)**	***p* value**	**Reference values**
**qBEI results: Trabecular (Tb) bone**				
**Bone mineralization density distribution (BMDD) parameter**				Age range: 2-20.9 yr, *n* = 50 (14)
**TbCaMean (weight % calcium)**	19.99 (18.99, 20.64)	21.50 (20.91, 22.29)^b^	.008	22.60 (22.12-22.96)
**TbCaPeak (weight % calcium)**	20.97 (20.10, 21.66)	22.53 (22.01, 23.22)^a^	.009	23.57 (23.01-23.78)
**TbCaWidth (Δ weight % calcium)**	5.63 (5.20, 6.59)	4.60 (4.51, 4.85)^c^	<.0001	3.64 (3.47-3.99)
**TbCaLow (% bone area)**	19.61 (15.84, 33.14)	10.01 (7.36, 11.78)^b^	.002	5.57 (4.78-6.80)
**TbCaHigh (% bone area)**	0.19 (0.12, 0.45)	0.91 (0.24, 1.72)	.089	1.52 (0.62-2.22)
**Osteocyte lacunar sections (OLS) parameter**				Age-range: 2-30 yr, *n* = 57 (16)
**TbOLS porosity (%)**	0.60 (0.51, 0.70)	0.55 (0.44, 0.58)	.169	0.54 (0.481-0.59)
**TbOLS density (number/mm**^**2**^**)**	231 (217.7, 254.6)	219.1 (185.3, 229.1)	.121	221.6 (204.3-243.9)
**TbOLS area (μm**^**2**^**)**	20.96 (20.96, 22.51)	20.96 (20.18, 22.51)	.613	20.18 (18.63-21.73)
**TbOLS perimeter (μm)**	20.24 (19.20, 20.75)	20.13 (19.51, 20.45)	.860	19.51 (18.62-20.31)
**TbOLS aspect ratio**	2.19 (2.09, 2.33)	2.25 (2.19, 2.33)	.279	2.30 (2.17–2.40)
**qBEI results: Cortical (Ct) bone**				
**Bone mineralization density distribution (BMDD) parameter**				Age range: 2-20.9 yr, *n* = 50 (14)
**CtCaMean (weight % calcium)**	20.39 (19.38, 21.44)	22.06 (21.31, 22.54)^a^	.046	22.17 (21.05-22.76)
**CtCaPeak (weight % calcium)**	21.02 (20.28, 22.62)	22.92 (22.18, 23.57)	.089	22.96 (22.10-23.48)
**CtCaWidth (Δ weight % calcium)**	5.81 (5.37, 6.24)	4.73 (4.33, 5.20)^b^	.005	4.07 (3.73-4.68)
**CtCaLow (% bone area)**	18.33 (12.04, 27.42)	8.44 (6.15, 11.78)^a^	.043	6.86 (5.06-11.48)
**CtCaHigh (% bone area)**	0.41 (0.12, 0.77)	1.11 (0.66, 2.99)^a^	.047	1.01 (0.44-1.89)
**Osteocyte lacunar sections (OLS) parameter**				Age-range: 2-30 yr, *n* = 57 (16)
**CtOLS porosity (%)**	0.63 (0.51, 0.81)	0.58 (0.53, 0.66)	.687	0.61 (0.54-0.72)
**CtOLS density (number/mm**^**2**^**)**	266.6 (240.8, 289.1)	261.8 (188.2, 274.9)	.598	262.3 (236.8-310.6)
**CtOLS area (μm**^**2**^**)**	20.96 (17.85, 23.28)	21.73 (17.85, 23.28)	1.000	18.63 (18.05-22.48)
**CtOLS perimeter (μm)**	19.61 (18.48, 20.24)	20.24 (18.48, 21.48)	.384	18.78 (17.64-20.65)
**CtOLS aspect ratio**	2.05 (1.94, 2.22)	2.17 (2.06, 2.38)	.078	2.24 (2.05-2.37)
**Results of immunohistochemistry/TUNEL analyses**				
**FGF23/B.Ar (#/mm** ^**2**^ **)**	0.7 (0.3, 11.2)	9.0 (1.0, 17.9)	.151	
**FGF23 (% tissue area)**	0.095 (0.075, 0.139)	0.151 (0.069, 0.213)	.521	
**FGF23 (staining intensity)**	0.823 (0.734, 0.866)	0.791 (0.740, 0.865)	.678	
**DMP1 (% tissue area)**	0.324 (0.274, 0.611)	0.522 (0.263, 0.710)	.427	
**DMP1 (staining intensity)**	0.862 (0.860, 0.884)	0.874 (0.848, 0.912)	.307	
**MEPE (% cells positive)**	6.1 (4.5, 10.6)	8.1 (5.9, 9.1)	.734	
**Sclerostin (% tissue area)**	0.077 (0.060, 0.177)	0.104 (0.078, 0.155)	.385	
**Sclerostin (staining intensity)**	0.880 (0.818, 0.922)	0.861 (0.827, 0.933)	.850	
**Bone TUNEL (% cells positive)**	4.6 (1.6, 17.3)	7.8 (3.1, 11.4)	.970	
**Marrow TUNEL (% cells positive)**	2.5 (0.9, 11.9)	7.4 (2.2, 9.4)	.623	

Data are reported as median with IQR. Reference values are reported as mean ± SE and median (range).

a *p* < .05 between high and normal/low turnover.

b *p* < .01 between high and normal/low turnover.

c *p* < .0001 between high and normal/low turnover.

**Table 3:** Correlations between biochemical values, bone protein expression, bone histomorphometric, and quantitative bone electron imaging parameters. Significant differences are displayed in bold.

	**BFR/BS**	**OV/BV**	**O.Th**	**TbCaMean**	**TbCaLow**	**TbOLS-Porosity**	**TbOLS-AR**	**CtCaMean**	**CtCaLow**	**CtOLS-porosity**	**CtOLS-AR**
**PTH**	0.44 0.04	**0.49** **0.02**	**0.63** **<0.01**	**−0.49** **0.02**	**0.57** **<0.01**	0.06 0.77	−0.23 0.30	−0.22 0.32	0.26 0.24	−0.06 0.78	−0.19 0.42
**Serum ALP**	**0.75** **<0.0001**	**0.90** **<0.0001**	**0.66** **<0.001**	**−0.83** **<0.0001**	**0.83** **<0.0001**	−0.07 0.75	**−0.49** **0.02**	**−0.67** **<0.001**	**0.73** **<0.0001**	0.28 0.20	**−0.44** **0.04**
**C-terminal FGF23**	−0.11 0.61	**−0.47** **0.02**	−0.01 0.98	0.32 0.13	−0.29 0.18	**0.53** **<0.01**	−0.04 0.85	0.23 0.29	−0.22 0.32	0.03 0.90	−0.08 0.71
**Intact FGF23**	−0.03 0.89	**−0.49** **0.03**	−0.03 0.91	0.28 0.23	−0.23 0.33	**0.45** **0.04**	0.17 0.47	0.25 0.28	−0.25 0.28	0.08 0.76	0.02 0.95
**FGF23/B/Ar**	−0.36 0.08	**−0.55** **<0.01**	**−0.42** **0.04**	**0.52** **<0.01**	**−0.50** **0.01**	0.10 0.64	0.30 0.16	**0.57** **<0.01**	**−0.54** **<0.01**	−0.04 0.86	**0.63** **<0.01**
**Sclerostin/Ar**	−0.28 0.23	−0.26 0.27	−0.32 0.17	0.39 0.09	−0.42 0.06	**−0.56** **<0.01**	0.40 0.08	**0.63** **<0.01**	**−0.58** **<0.01**	**−0.64** **<0.01**	0.36 0.12
**TUNEL**	0.03 0.90	0.07 0.76	−0.02 0.92	0.10 0.68	−0.11 0.64	**−0.52** **0.02**	0.22 0.35	0.40 0.08	−0.29 0.22	**−0.66** **<0.01**	0.33 0.16
**DMP1**	−0.25 0.29	−0.12 0.63	−0.16 0.49	0.13 0.60	−0.17 0.49	0.05 0.85	−0.06 0.81	0.22 0.35	−0.09 0.70	0.04 0.88	0.02 0.94
**MEPE**	−0.07 0.78	0.03 0.91	0.07 0.77	−0.15 0.52	0.09 0.69	−0.03 0.90	−0.08 0.72	−0.06 0.81	0.15 0.52	−0.08 0.75	−0.24 0.33
**BFR/BS**	1.00	**0.77** **<0.0001**	**0.70** **<0.001**	**−0.72** **<0.0001**	**0.76** **<0.0001**	<0.01 0.99	−0.33 0.11	**−0.54** **<0.01**	**0.62** **<0.01**	0.21 0.34	−0.33 0.13
**OV/BV**		1.00	**0.67** **<0.001**	**−0.77** **<0.0001**	**0.78** **<0.0001**	−0.23 0.27	−0.31 0.14	**−0.58** **<0.01**	**0.67** **<0.001**	0.10 0.64	−0.31 0.16
**O.Th**			1.00	**−0.68** **<0.001**	**0.71** **0.0001**	−0.03 0.88	−0.15 0.50	**−0.50** **0.01**	**0.56** **<0.01**	0.03 0.91	**−0.44** **0.04**
**TbCaMean**				1.00	**−0.99** **<0.0001**	0.01 0.97	**0.45** **0.03**	**0.82** **<0.0001**	**−0.86** **<0.0001**	−0.12 0.59	0.37 0.08
**TbCaLow**					1.00	<0.01 0.99	**−0.41** **0.04**	**−0.78** **<0.0001**	**0.82** **<0.0001**	0.09 0.69	−0.35 0.10
**TbOLS-Porosity**						1.00	−0.24 0.27	−0.27 0.20	0.19 0.37	**0.44** **0.04**	−0.26 0.23
**TbOLS AR**							1.00	**0.51** **0.01**	**−0.57** **0.01**	**−0.56** **<0.01**	**0.42** **<0.05**
**CtCaMean**								1.00	**−0.96** **<0.0001**	−0.28 0.20	**0.46** **0.03**
**CtCaLow**									1.00	0.31 0.15	**−0.47** **0.02**
**CtOLS-Porosity**										1.00	−0.41 0.06

By histomorphometry, 8 (33%) of samples had an increase in osteoid thickness (O.Th), 29% had an increase in osteoid volume (OV/BV), and 17% samples were classified as having a true mineralization defect (osteomalacia) based on an increase in osteoid thickness (O.Th > 10 μm) in conjunction with prolonged MLT (MLT > 27.1 d). These samples (shown as triangles in Figure 1A-H) also exhibited the highest osteoid volumes (OV/BV) of the total cohort. Three of them were classified has having high BFR/BS while BFR/BS was normal in one (shown as white and black triangles respectively in Figure 1). By contrast, qBEI identified a much larger proportion of samples (88%) with an above normal percentage of bone in primary mineralization (defined by TbCaLow > 6.8% bone area) (Figure 1A and C). In addition, 75% of samples had abnormally low average mineralization, (defined by a TbCaMean < 22.12 wt% calcium, (Figure 1B and D). Hypomineralization of the bone matrix was also detected in cortical bone. CtCaLow was increased (>11.5% bone area) in 48% of samples and CtCaMean was decreased (<21.05 wt% calcium) in 30% of the samples (data not shown). Of note, these percentages did not include the one biopsy sample with osteomalacia and low/normal BFR/BS which did not contain enough cortical bone for evaluation. Dialysis vintage was not related to the prevalence of defective matrix mineralization, either as detected by histomorphometry or by qBEI.

Trabecular bone volume, including both the mineralized matrix and unmineralized osteoid, was measured as BV/TV on bone histomorphometry. BV/TV was within normal age limits (*n* = 13) or above normal (*n* = 10) in all patients except for one whose bone volume was slightly below the reference range observed in healthy individuals.^11^ Mineralized trabecular bone volume (Md.BV/TV) was measured by qBEI alone and was also normal or slightly above normal values in most patients (Figure 1G and H).

### Osteocyte lacunar size and density are normal in ESKD bone; however, osteocyte configuration is immaturely rounded when bone matrix is poorly mineralized

Altered osteocyte lacunar size and density are features of bone in patients with normal kidney function who have abnormal bone matrix mineralization, including patients with osteogenesis imperfecta,^14^ patients receiving teriparatide^19^^,^^20^ and patients with hypophosphatemic rickets.^21^^,^^22^ In addition, OLS porosity and density have been related to secondary hyperparathyroidism in adult CKD bone.^23^ We thus evaluated characteristics of osteocyte lacunar sections (OLS) by qBEI in the current cohort. The average OLS size (area and perimeter) as well as the OLS porosity, density, and aspect ratio were within normal reference ranges (16) (see Table S2) and did not differ between samples with high and those with low/normal BFR/BS (Table 2). However, bone samples from patients who had been treated with maintenance dialysis for over 2 yr prior to bone biopsy demonstrated greater trabecular OLS porosity (*p* = .03) and greater trabecular OLS density (*p* = .02) than did samples from patients with less cumulative time on dialysis (data not shown). In addition, although overall values for osteocyte aspect ratio did not differ in ESKD samples relative to control values, the measure of elongation of the lacunae in cortical bone, defined by the aspect ratio (CtOLS-AR), was abnormally low (ie, lacunae were inappropriately rounded) in 8 samples. CtOLS-AR values correlated directly with CtCaMean and inversely with CtCaLow and TbCaLow (Table 3), suggesting that bone with more elongated osteocyte lacunar conformation also had denser mineral content.

### Higher bone FGF23-expression associates with milder mineralization defects and an appropriately elongated osteocyte lacunar shape

**Figure 2:**
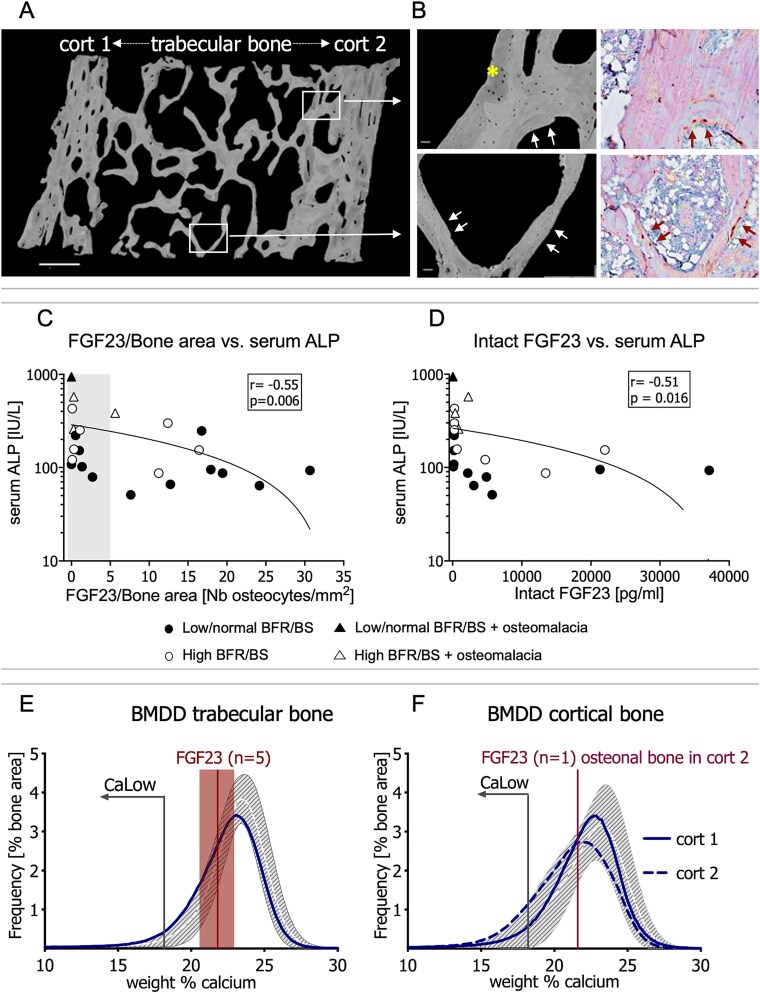
Alignment of qBEI and FGF23 stained sections. (A) Quantitative backscattered electron microscopy image of a representative sample of iliac crest bone sample showing both the trabeculae and 2 cortices (cort 1 and cort 2). Gray represents the mineralized bone matrix: brighter gray represents higher mineralized matrix, darker gray represents lowly mineralized matrix (yellow asterisk Figure 1B), black represents unmineralized bone (ie, osteocyte lacunae and bone marrow). Windowed areas are enlarged in (B) where the immunohistochemical detection of FGF23-expressing osteocytes (red arrows) is also shown. White arrows show corresponding regions on the qBEI images. (C) Serum ALP as a function of bone FGF23 expression (FGF23/bone area; immunohistochemistry). (D) Serum ALP as a function of serum intact FGF23. Bone mineralization density distribution (BMDD) curved of trabecular (E) and cortical (F) bone of the sample shown in (A). The gray shading represents the pediatric reference BMDD curve (median (25th, 75th IQR) from Mähr et al.^14^ The red shading/bar represents the calcium content of bone packets containing FGF23-expressing osteocytes (in this sample 5 bone packets were viewed in trabecular and 1 bone packet in cortical bone). Note that in both bone compartments, the blue line (representing the BMDD of the patient) is slightly shifted to the left (ie, toward lower mineralization density) as compared to the mean reference value in healthy adolescents (white dashed line). The vertical axes in panels C and D are on a logarithmic scale.

In line with our previous reports,^9^^,^^10^ we found that bone packets containing FGF23-expressing osteocytes were at the periphery of trabecular and cortical bone. A representative bone section, demonstrating the immunohistochemical detection of FGF23 and the quantification of calcium in the surrounding bone packets, is shown in Figure 2A and B. Sixty-three percent of samples (*n* = 15) contained at least one clearly identifiable bone packet containing an FGF23-expressing osteocyte. Fifty percent of samples (*n* = 12) had numbers of FGF23-expressing osteocytes that were above those previously observed in healthy control samples (*>*5/mm^2^)^9^ (see Table S4). Patient age did not differ between samples with high and those with low/normal numbers of FGF23-expressing osteocytes (*p* = .16). The coefficient of variation in numbers of FGF23-expressing osteocytes per bone area was 112% among samples while the intensity of FGF23 expression varied only 15% (data not shown).

Numbers of FGF23-expressing osteocytes correlated directly with circulating intact, but not C-terminal, FGF23 values (intact: *r* = 0.55, *p* = .013; C-terminal: *r* = 0.36, *p* = .09). Numbers of FGF23-expressing osteocytes correlated directly with TbCaMean and CtCaMean obtained by qBEI and inversely with TbCaLow, CtCalow, and histomorphometric parameters of osteoid accumulation (Table 3). Numbers of FGF23-expressing osteocytes, as well as the values of circulating intact FGF23, correlated inversely correlated with circulating ALP (*r* = −0.55, *p* = .006; *r* = −0.51, *p* = .0016, respectively) (Figure 2C and D). The relationship between bone FGF23 expression and ALP, however, was complex. Very low bone FGF23 expression (as well as very low circulating FGF23 concentrations) associated with high ALP levels. Thus, the 4 samples with osteomalacia all displayed low bone FGF23 expression and low circulating FGF23 concentrations. Low serum ALP levels, by contrast, associated with a wide range of FGF23-expressing osteocytes and circulating FGF23 levels (Figure 2C and D). Numbers of FGF23-expressing osteocytes were low (FGF23/B.Ar: 0.3 (0.1, 0.7)) in bone samples with inappropriately rounded cortical osteocyte lacunae (CtOLS-AR values below reference values). By contrast, bone FGF23/B.Ar was 10.8 (2.4, 16.5) (*p* = .018 between groups) in samples with normally elliptical cortical osteocyte lacunae (CtOLS-AR values within reference range).

**Figure 3:**
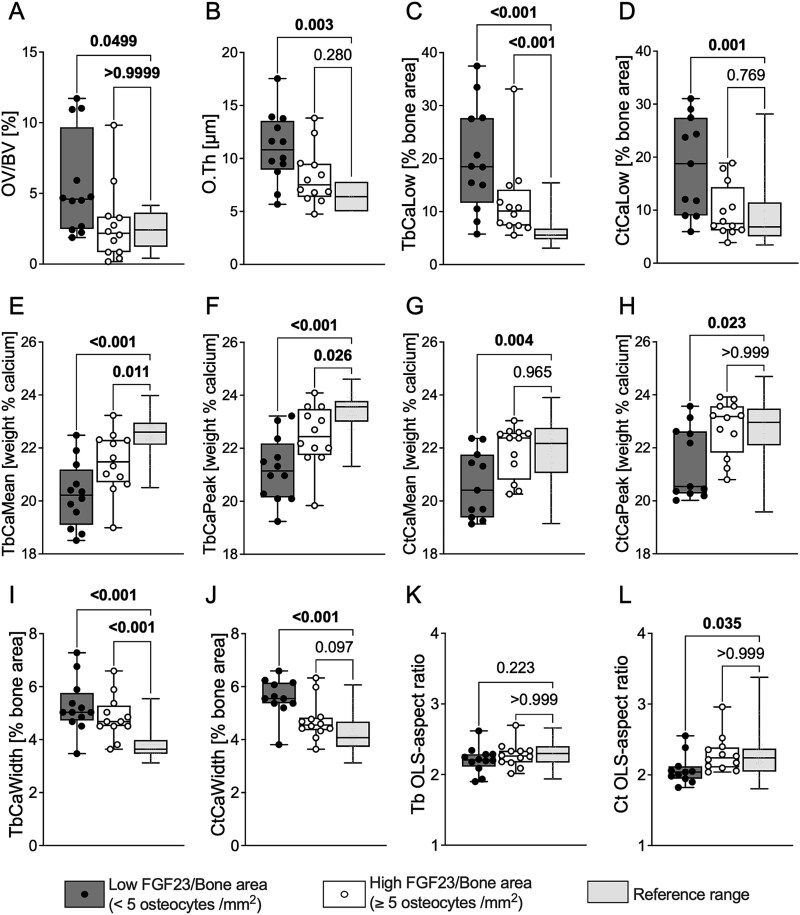
Comparison of bone histomorphometric and qBEI measures of mineralization along with osteocyte lacunar shape (OLS-aspect ratio) in samples with low bone FGF23 expression (<5 osteocytes/mm^2^; dark gray boxes/black circles) and high bone FGF23 expression (*>*5 osteocytes/mm^2^; white boxes/white circles) versus reference range in healthy adolescents (light gray boxes). (A) Osteoid volume (OV/BV). (B) Osteoid thickness (O.Th). (C and D) Percentage of lowly mineralized matrix in trabecular bone (TbCaLow) and in cortical bone (CtCaLow). (E and F) Mean and peak calcium content in trabecular bone (TbCaMean and TbCaPeak). (G and H) Mean and peak calcium content in cortical bone (CtCaMean and CtCaPeak). (I and J) Heterogeneity of mineralization in trabecular (TbCaWidth) and cortical (CtCaWidth) bone. (K and L) Osteocyte lacunar shape (OLS-aspect ratio) in trabecular (Tb) and cortical (Ct) bone. Note that all parameters are closer to reference values in the group of FGF23/bone area *>*5 osteocytes/mm^2^. In particular, O.Th, CtCaLow, CtCaMean, CtCaPeak, and CtOLS-AR are shifted toward normal range in samples with high bone FGF23 expression. Boxes represent median values (25th, 75th IQR); whiskers extend from minimal to maximal values. Single data points are shown as circles. Reference values for BMDD parameters from Mähr et al.,^14^ for bone histomorphometry parameters from Glorieux et al.^11^; and for FGF23/bone area from Pereira et al.^9^

Since numbers of FGF23-expressing osteocytes correlated with matrix mineralization parameters but not with bone formation rates (Table 3), we hypothesized that some defect in osteocyte function contributes to impaired matrix maturation in a way that is independent of bone formation upon the bone surface. We first characterized the local environment surrounding FGF23-expressing osteocytes. While the calcium content of bone packets containing FGF23-expressing osteocytes varied from sample to sample, their calcium contents were consistently close to the most frequent calcium content (CaPeak) of the specific sample being measured (Figure 2E and F). This suggested that the bone matrix surrounding FGF23-expressing osteocytes, although in an early phase of secondary mineralization, had rapidly accumulated mineral. We next compared surface (histomorphometric) osteoid indices and matrix (qBEI) BMDD parameters in samples with low versus high numbers of FGF23-expressing osteocytes. As shown in Figure 3A-H, OV/BV, O.Th, TbCaLow, and CtCaLow were significantly higher (while values of TbCaMean, TbCaPeak, CtCaMean, and CtCaPeak were lower) than reference values in samples with low numbers of FGF23-expressing osteocytes. By contrast, OV/BV, O.Th, CtCaLow, CtCaMean, and CtCaPeak were all within normal reference ranges in biopsy samples with high numbers of FGF23-expressing osteocytes. TbCaLow was higher, while TbCaPeak and TbCaMean were lower (and closer to reference values), in samples with high numbers of FGF23-expressing osteocytes (Figure 3C, E, and  F). As a result, the heterogeneity of mineralization (CaWidth) was increased in both trabecular and cortical compartments of samples with low numbers of FGF23-expressing osteocytes (Figure 3I, J). By contrast, TbCaWidth was closer to and CtCaWidth fell within reference ranges in samples with high numbers of FGF23-expressing osteocytes. Normally elongated osteocyte lacunar conformation (OLS-AR) was observed in both trabecular and cortical compartments of samples with high, while a more rounded conformation was observed in cortical samples with low/normal numbers of FGF23-expressing osteocytes (Figure 3K and L). No other osteocyte parameters associated with numbers of FGF23-expressing osteocytes and all fell within reference ranges (data not shown). Together, these data suggest that FGF23-expressing osteocytes are found in more mature bone that has experienced a more efficient accumulation of mineral.

**Figure 4:**
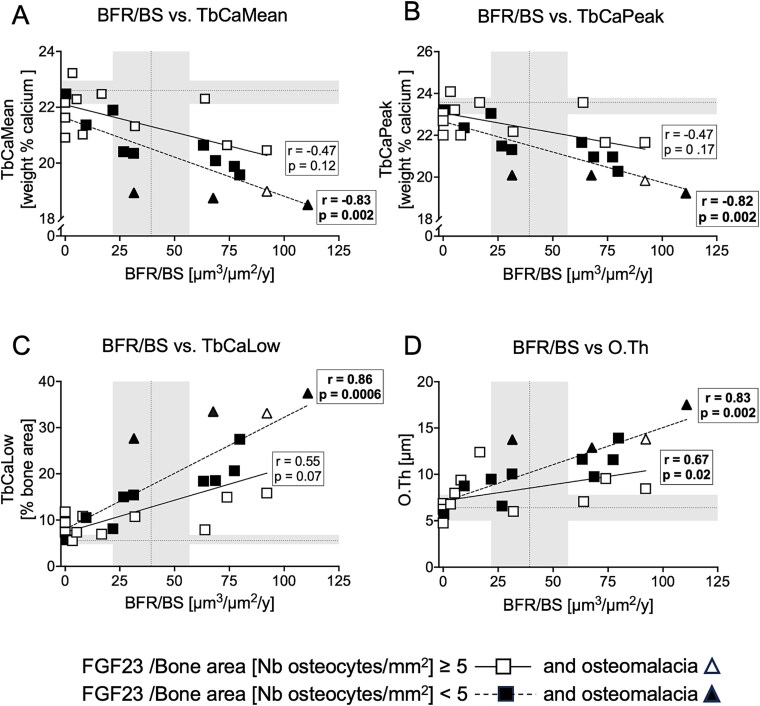
The relationship between trabecular bone formation rates (BFR/BS) and bone mineralization is modified by the presence of FGF23-expressing osteocytes. (A) Trabecular bone matrix mineralization (TbCaMean), (B) most frequently observed calcium content in trabecular bone (TbCaPeak), (C) percentage of lowly mineralized trabecular bone area (TbCaLow), and (D) osteoid thickness (O.Th). Open symbols: high bone FGF23 expression (FGF23/bone area *>* 5/mm^2^); closed symbols: low/normal bone FGF23 expression (FGF23/bone area < 5/mm^2^); triangles: samples with osteomalacia. Note that in the low/normal bone FGF23 expressing group, there is an inverse correlation between BFR/BS and TbCaMean and TbCaPeak (dashed line), whereas the significance is lost in patients with high bone FGF23 expression (continuous line). Thus, the higher degree of bone matrix mineralization in the high bone FGF23 expressing group does not strictly depend on BFR/BS measured on bone surfaces. Conversely, there is a direct correlation between BFR/BS and TbCaLow and O.Th. in the low/normal bone FGF23 expressing group which is attenuated in the high FGF23 expressing group these data suggest a positive role for FGF23 for bone matrix mineralization beyond the effects of BFR/BS. Normal ranges of bone FGF23 expression were previously reported.^9^ Gray bands represent reference ranges in healthy children. Histomorphometric reference values are from Glorieux et al.^11^; pediatric bone mineralization density distribution (BMDD) reference values (median (25th, 75th IQR) are from Mähr et al.^14^

We next evaluated whether the relative abundance or paucity of FGF23-expressing osteocytes was related to any variation in the relationship between surface bone formation and bone matrix mineralization. While surface osteoid indices and matrix mineralization parameters associated with bone formation rates, the correlation was stronger in samples with low numbers of FGF23-expressing osteocytes (Figure 4A-D). For a given bone formation rate, TbCaMean, TbCaPeak were generally higher—while TbCaLow and osteoid accumulation were lower—when numbers of FGF23-expressing osteocytes were high. Multivariable analysis, shown in Table S3, confirmed that the presence of FGF23-expressing osteocytes modified the relationship between bone formation and bone matrix mineralization. High numbers of FGF23-expressing osteocytes were associated with improved mineralization at every rate of bone formation.

We then evaluated whether other osteocyte-specific proteins, including sclerostin (a marker of mature osteocytes that also functions as a Wnt signaling inhibitor^7^^,^^24^), DMP1 and MEPE (2 small integrin binding ligand N-linked glycoproteins (SIBLINGs) that regulate bone cell maturation and bone matrix mineralization) and osteocyte apoptosis (TUNEL staining), were related to bone surface and matrix mineralization. Consistent with its presence in mature osteocytes, sclerostin expression correlated with the amount of TUNEL expression (*r* = 0.80, *p* < .0001) and staining for each correlated inversely with cortical and cancellous bone porosity. Sclerostin expression also followed FGF23 expression, demonstrating a direct correlation with mean trabecular calcium content (TbCaMean) (Table 3). DMP1 was expressed more widely than FGF23 and was identified throughout bone matrix. DMP1 was observed in all samples. Similar intensities of expression were observed in DMP1 among samples, with a coefficient of variation of less than 10%. DMP1 expression did not correlate with FGF23 expression. The amount and intensity of DMP1 expression did not correlate with biochemical, bone histomorphometric, and qBEI parameters. Matrix extracellular phosphoglycoprotein was expressed in osteocytes deep in trabeculae in all samples and, like DMP1, its expression did not correlate with expression of FGF23 or with biochemical, bone histomorphometric, of qBEI parameters. Thus, while expression of these bone proteins has known effects on the maturation of osteoblasts and osteocytes, apart from FGF23, only sclerostin and TUNEL staining had any relationship to physical measures of bone quality.

## Discussion

In this manuscript, we demonstrate that bone matrix mineralization is impaired in adolescents with ESKD, including patients with low, normal, and high trabecular bone turnover. We also demonstrate that traditional histomorphometric measures of unmineralized osteoid accumulation, while correlating with bone matrix mineralization parameters obtained by qBEI, underestimate the prevalence of bone matrix under-mineralization. Finally, we demonstrate that the presence of high numbers of FGF23-expressing osteocytes signals more appropriate underlying matrix maturation and bone mineralization characteristics than is found in samples with low numbers of FGF23-expressing osteocytes.

Bone matrix mineralization is a biphasic process. Once started, mineral content of newly formed osteoid rapidly increases within a few days up to 60%-70% of its final value.^25^ This initial phase of primary mineralization is represented by the percentage of bone that is mineralized below the fifth percentile of adult reference values (reported as the parameter CaLow on qBEI).^15^^,^^26^ An increase in bone undergoing primary mineralization can arise from elevated bone formation, during which high numbers of new bone packets are simultaneously formed; from abnormal mineralization kinetics; or from a combination of the two.^26^ Skeletal growth is associated with elevated bone formation.^27^ Thus, appropriate, age-specific, reference ranges for BMDD parameters (as were used in the current study^14^) are critical when interpreting bone matrix mineralization in children and adolescents. Even accounting for growth, the proportion of lowly mineralized bone was increased in the current cohort. Importantly, excess CaLow was observed not only in bone cores with high rates of bone formation but also in most cores classified as low-to-normal bone turnover. This suggests that a large proportion of pediatric and adolescent patients with ESKD have impaired primary mineralization kinetics that are not detected by conventional histomorphometry, which distinguishes solely between mineralized and unmineralized matrix (osteoid) on the bone surface. Our present observations are further consistent with data showing a marked increases in lowly mineralized bone matrix in bone of adult CKD patients.^23^ Secondary mineralization, a slow process of mineral accrual which follows primary mineralization, also appears impaired in our patient cohort since the most frequent calcium content (CaPeak) as well as the overall mineralization status of the bone samples (CaMean) were both abnormally low, even in patients with low/normal BFR/BS. In the current study, 75% of samples had average trabecular matrix mineralization (TbCaMean) that was below normal limits.

Despite the high prevalence of bone under-mineralization, osteocyte size (perimeter and area) and density were within normal limits in the current cohort^16^ and did not vary with PTH (data not shown). This contrasts with the increased osteocyte lacunar size and the presence of hypomineralized periosteocytic lesions that are found in patients and animal models with normal kidney function who have rickets/osteomalacia due to dietary calcium deficiency^28^ or hypophosphatemia^21^^,^^22^^,^^29^ many of whom also have excess circulating FGF23.^30^^,^^31^ However, cortical osteocyte lacunar aspect ratio was inappropriately low in many samples from our cohort, signifying a more rounded lacunar shape and possibly also a stiffer cytoskeletal configuration that responds poorly to mechanical stimuli in vivo*.*^32–34^ Since osteocyte lacunae orient themselves in parallel to the collagen lamellae, elongated lacunae reflect more mature lamellar bone while rounder lacunae characterize a less organized bone matrix.^35^ The presence of more rounded osteocyte lacunae is indicative of a defect in matrix organization (particularly in the cortex) in ESKD bone. Because cortical bone contributes to about 80% of skeletal mass and because cortical bone sustains most of the weight-bearing load, inappropriate mineral content (particularly in the context of high bone formation rates) and deficits in matrix organization in FGF23-poor CKD bone may have major impacts on the biomechanical competence of bone.^36^

We have previously shown that FGF23-expressing osteocytes are embedded in bone that is in early secondary mineralization.^10^ We demonstrate here that the density of these newly mineralized, FGF23-expressing, bone packets varies considerably and their calcium content is often much lower than the typical calcium content (CaPeak) observed in biopsy samples of healthy controls.^14^ However, their calcium content is typically close to—and often slightly higher than—the CaPeak in the sample being measured. Importantly, CaPeak was higher, and the heterogeneity of mineralization (CaWidth) was narrower, and both parameters were close to reference values in ESKD samples with high FGF23 expression. These unexpected findings suggest that ESKD samples with higher numbers of FGF23-expressing osteocytes have a more tightly coordinated matrix mineralization than do samples with lower FGF23 expression. The co-incidence of excess bone FGF23 and impaired bone mineralization in patients and animals with normal kidney function^30^^,^^31^^,^^37^ has contributed to the traditional dogma that FGF23 directly impairs bone mineralization.^38^ The direct local effect of FGF23 on bone, however, is difficult to evaluate since FGF23 excess also results in systemic hypophosphatemia, which itself prevents normal bone mineralization. It is well accepted that ALP expression precedes FGF23 expression during osteocyte differentiation.^39^ Alkaline phosphatase promotes bone matrix mineralization by cleaving the mineralization inhibitor pyrophosphate (PPi), providing the inorganic phosphate that, in conjunction with calcium and in the presence of SIBLING proteins, such as osteopontin, regulates nucleation of hydroxyapatite in the extracellular matrix.^40^^,^^41^ In the absence of ALP, severe mineralization defects occur.^42^^,^^43^ Continuous or prolonged expression of ALP, however, particularly when osteoblast to osteocyte maturation is impaired, may increase local expression of the mineralization inhibitor osteopontin, which in turn prevents appropriate local mineralization. In this complex interplay, FGF23, which is expressed by osteocytes which have completed primary mineralization, may serve to counter the effects of ALP. Interestingly, previous studies have suggested that FGF23 not only inhibits local ALP activity, allowing PPi to accumulate^44^ but, in contrast to ALP, indirectly downregulates OPN secretion and thus prevents the local accumulation of osteopontin.^44^^,^^45^ It is noteworthy that in patients with normal kidney function who have a primary FGF23 excess (ie, patients with X-linked hypophosphatemia), bone matrix is not generally under-mineralized but rather consists of normally (or highly) mineralized bone matrix interspersed with patches of lowly mineralized bone and non-mineralized osteoid.^21^^,^^29^ Demineralization of the perilacunar matrix (osteocytic osteolysis)^20^^,^^22^ may also be present but inherent defects in secondary mineralization of bone matrix are not prevalent since CaPeak is within—or even above—normal limits.^21^^,^^29^^,^^46^ Moreover, the lack of PHEX in XLH results in a local accumulation of its substrate osteopontin within the bone matrix that might directly impact the normal mineralization process.^41^

The factors that influence bone matrix maturation and mineralization remain incompletely understood—and this is particularly true in CKD bone in which the phenomenon of skeletal PTH resistance (the pathophysiology of which is only partially understood) complicates the relationship between PTH and bone.^47^ In the context of ESKD, where renal phosphate wasting is absent, nature provides a situation in which FGF23 excess and matrix mineralization defects co-exist, while circulating calcium and phosphate concentrations are adequate (and often excessive), allowing for unique observations into the relationship between local bone FGF23 expression and local bone mineralization. Patients in our cohort with high bone FGF23 consistently displayed low circulating ALP concentrations. Similarly, patients with high circulating ALP (including the samples diagnosed with osteomalacia by bone histomorphometry) consistently displayed low bone FGF23 expression in conjunction with low bone matrix mineralization. This is consistent with a negative feedback loop in which FGF23 expression, reflecting appropriate osteocyte transition and matrix mineralization, limits further PPi exposure and ALP expression.^41^^,^^48^^,^^49^ A subset of our cohort, however, displayed both low bone FGF23 expression and low circulating ALP. This suggests a subgroup of patients in whom osteoblast and osteocyte maturation are severely impaired, resulting in severe osteomalacia.

Higher numbers of FGF23-expressing osteocytes in biopsy samples with higher bone matrix mineralization suggest that higher FGF23 expression might be present in highly mineralized adynamic bone—that is, bone with very low bone formation rates. However, stagnantly adynamic bone does not produce new bone, which is essential for forming packets of bone in an early stage of secondary mineralization—that is, the packets of bone surrounding FGF23-expressing osteocytes. Although there were no differences in bone FGF23 expression in patients with high versus those with low and normal bone formation rates, the presence of FGF23 was a significant modifying factor when considering the effects of bone formation on skeletal mineralization. Previous studies have demonstrated that defective bone mineralization is observed in CKD patients with all rates of bone formation,^50^ suggesting a unique disconnect between bone turnover and mineralization in CKD bone. Our finding that numbers of FGF23-expressing osteocytes modify the relationship between bone turnover, osteoid mineralization, and matrix mineralization highlights the importance of CKD as a disease model in which the relationship between FGF23 and bone can be defined, independent of circulating phosphate.

We observed very little correlation between circulating calcium, phosphorus, and PTH concentrations and the number of FGF23-expressing osteocytes in bone. Moreover, only subtle differences in FGF23 staining intensity were observed between osteocytes. This suggests that there is a plateau in FGF23 expression by individual osteocytes in bone and that the ability of an osteoblast to transition to an osteocyte phenotype has direct feedback on its own local environment—a feedback that appears to be more powerful than the quantity of FGF23 expression by individual cells. It is important to acknowledge, however, that we do not know whether other factors (such as FGF23-modifiers including GALNT3 or FAM20C) affect FGF23 expression CKD bone. In addition, we do not know whether increased expression of FGF23 from bone alone is the cause of increased circulating FGF23 levels in CKD. Others have demonstrated that this may not be the case. Fibrotic liver and hypertrophied hearts, for instance, also contribute to circulating FGF23 levels and may play a role in elevated values in CKD.^51^ Thus, while modest correlations were observed between numbers of FGF23-expressing osteocytes and circulating FGF23 concentrations, the use of circulating FGF23 concentrations in the prediction of bone maturation and mineralization requires further study.

We acknowledge that the current study has certain limitations. This analysis was performed on tissue obtained from pediatric patients; more specifically, nearly all the samples came from adolescent patients. While we do not know if all of these findings can be generalized to the adult population, it is interesting to note that increased numbers of FGF23-expressing osteocytes have been observed in adult pre-dialysis CKD and dialysis patients^5^^,^^9^ and circulating FGF23 levels and bone FGF23 expression correlate inversely with mineralization rates in children and adults alike.^9^^,^^52^ Moreover, previous adult data have shown a similar increase in the amount bone in primary mineralization and a similar increase in the amount of under-mineralized bone in secondary mineralization.^23^ However, the interactions between CKD-mediated osteocyte maturation impairment, numbers of early osteocytes, and bone mineralization, also outside the pediatric age range warrant further investigation. In addition, the vast majority of samples in this study were from patients who had received vitamin D sterols in their past treatment. This treatment may have affected bone FGF23 expression and may also have affected bone cell maturation, collagen deposition, and bone matrix mineralization. Regardless of treatment received, however, the relationship between bone protein expression and the maturity of bone remains. Finally, the reference cohorts cited for histomorphometry were from Glorieux et al.^11^ Reference cohorts for healthy children, adolescents, and young adults are scarce and largely historical, due to ethical constraints on recruiting healthy children as control subjects for bone biopsy studies. The current study references the Glorieux controls because these controls represent the largest cohort of healthy children that has been published so far and because the samples from this data set were used to establish the qBEI references used in this study, allowing exact categorization of histomorphometric, BMDD, and osteocyte lacunar parameters as normal or outside the normal range.

In conclusion, matrix mineralization defects are highly prevalent—and underappreciated—in CKD bone. These defects occur even when histomorphometric analyses of osteoid accumulation and mineralization rates are normal. Clinical bone disease—including increased fracture rates—is prevalent in CKD patients and the high prevalence of under-mineralized bone identified in this study may contribute to poor bone quality in this population. The ability of bone to maintain increased numbers of FGF23-expressing osteocytes, may reflect a physiologic response to the toxic effects of uremia on bone cell maturation and mineralization that has been previously described.^10^

## Supplementary Material

3_3_26_supplemental_materia_ziag036

fig9new_ziag036

Slide9_ziag036

## Data Availability

Quantitative backscattered electron imaging (qBEI) data (BMDD and OLS) supporting this publication can be accessed at our institutional digital data repository for published research via creed.lbg.ac.at. Biochemical and bone histomorphometric data from this publication can be requested through the lead contact, but is subject to required site specific approvals.
