# Supplementary material for: Impaired bone matrix maturation and mineralization are prevalent in adolescent end-stage kidney disease
Source: JBMR Plus. 2026 Mar 12;10(5):ziag036. doi: 10.1093/jbmrpl/ziag036 (PMC13050508; doi:10.1093/jbmrpl/ziag036)
Supplement: 3_3_26_supplemental_materia_ziag036 [file 3_3_26_supplemental_materia_ziag036.docx]

**Supplemental Figure 1:** Study design.

**Supplemental Table 1.** Demographic, biochemical, and bone histomorphometric values in the entire ESKD cohort.

| **Demographics** | |  |
| --- | --- | --- |
| Age (years) | 17.4 (15.2, 19.7) |  |
| Sex (M/F) | 17/7 |  |
| Underlying disease (n)  GN  CAKUT  Other | 6  10  8 |  |
| **Biochemicals** | | Normal ranges |
| Calcium (mg/dL) | 9.2 (8.6, 9.4) | 8.4 - 10.2 |
| Phosphorus (mg/dL) | 6.1 (5.0, 8.5) | age specific |
| ALP (IU/L) | 152 (87, 259) | Age and lab specific |
| PTH (pg/mL) | 534 (247, 1046) | 10 - 65 |
| Intact FGF23 (pg/mL) | 1527 (241, 5300) | <100 |
| C-terminal FGF23 (RU/mL) | 1989 (460, 5577) | <100 |
| **Trabecular bone histomorphometry** | | Normal ranges (11) |
| **Turnover** | | |
| Bone formation  (BFR/BS; um^3^/µm^2^/yr) | 31.4 (6.6, 71.3) | 39.3 ± 17.5  35.5 (6.8 - 78.4) |
| Eroded surface  (ES/BS; %) | 10.4 (4.4, 13.5) | 16.6 ± 5.6  15.6 (8.5 - 32.5) |
| **Mineralization** | | |
| Osteoid surface  (OS/BS; %) | 28.7 (15.9, 36.8) | 24.9 ± 10.0  22.5 (4.9 - 54.3) |
| Osteoid volume  (OV/BV; %) | 3.0 (2.0, 5.3) | 2.4 ± 1.2  2.26 (0.41- 4.15) |
| Osteoid thickness  (O.Th; µm) | 9.4 (6.8, 12.0) | 6.4 ± 1.4  6.1 (3.9 - 10) |
| Mineralization lag time (MLT; d) | 28.2 (20.2, 55.1) | 15.5 ± 4.8  15. 1 (8.7 - 29.1) |
| Osteoid maturation time (OMT; d) | 13.4 (9.6, 17.4) | 7.3 ± 1.9  6.9 (4.4 - 11.9) |
| **Volume** | | |
| Bone volume (BV/TV; %) | 31.9 (26.3, 38.9) | 23.3 ± 5.3  24.0 (13.5 - 35.5) |
| Mineralized bone volume (Md.BV/TV; %) | 31.1 (25.8, 36.0) | 23.3 ± 5.4  23.3 (12.9 - 35.1) |

Data are reported as median (interquartile range).

Reference values are reported as mean +/- SD and median (interquartile range)

**Supplemental Table 2.** Bone matrix material properties and bone protein expression in the entire ESKD cohort.

| **Quantitative Backscattered Electron Imaging (qBEI):**  **Bone mineralization density distribution (BMDD) and osteocyte lacunar sections (OLS)** | | | |
| --- | --- | --- | --- |
| **Trabecular (Tb) bone** (n=24) | | Normal range* | Difference (P value) |
| **BMDD parameter** |  | n=50 (14) |  |
| TbCaMean (weight % calcium) | 20.78 (19.99, 22.03) | 22.60 (22.12 - 22.96) | <0.0001 |
| TbCaPeak (weight % calcium) | 21.84 (20.97, 23.05) | 23.57 (23.01 - 23.78) | <0.0001 |
| TbCaWidth (Δ weight % calcium) | 4.94 (4.51, 5.37) | 3.64 (3.47 - 3.99) | <0.0001 |
| TbCaLow (% bone area) | 13.36 (8.00, 19.61) | 5.57 (4.78 - 6.80) | <0.0001 |
| TbCaHigh (% bone area) | 0.44 (0.16, 1.26) | 1.52 (0.62 - 2.22) | 0.0010 |
| **OLS parameter** |  | n=57 (16) |  |
| TbOLS porosity (%) | 0.56 (0.49, 0.65) | 0.54 (0.481 - 0.59) | 0.33 |
| TbOLS density (number/mm^2^) | 222.6 (210.1, 237.9) | 221.6 (204.3 - 243.9) | 0.96 |
| TbOLS area (µm^2^) | 20.96 (20.18, 22.51) | 20.18 (18.63 - 21.73) | 0.055 |
| TbOLS perimeter (µm) | 20.24 (19.36, 20.60) | 19.51 (18.62 - 20.31) | 0.07 |
| TbOLS aspect ratio | 2.24 (2.17, 2.33) | 2.30 (2.17 - 2.40) | 0.16 |
| **Cortical (Ct) bone** (n=23) | |  |  |
| **BMDD parameter** |  | n=48 (14) |  |
| CtCaMean (weight % calcium) | 21.44 (20.26, 22.38) | 22.17 (21.05 - 22.76) | 0.07 |
| CtCaPeak (weight % calcium) | 22.53 (20.54, 23.22) | 22.96 (22.10 - 23.48) | 0.21 |
| CtCaWidth (Δ weight % calcium) | 5.20 (4.42, 5.98) | 4.07 (3.73 - 4.68) | <0.0001 |
| CtCaLow (% bone area) | 10.60 (7.05, 18.88) | 6.86 (5.06 - 11.48) | 0.006 |
| CtCaHigh (% bone area) | 0.77 (0.36, 1.63) | 1.01 (0.44 - 1.89) | 0.56 |
| **OLS parameter** |  | n=57 (16) |  |
| CtOLS porosity (%) | 0.58 (0.51, 0.74) | 0.61 (0.54 - 0.72) | 0.64 |
| CtOLS density (number/mm^2^) | 264.9 (211.6, 287.4) | 262.3 (236.8 - 310.6) | 0.34 |
| CtOLS area (µm^2^) | 20.96 (17.85, 23.28) | 18.63 (18.05 - 22.48) | 0.31 |
| CtOLS perimeter (µm) | 19.81 (18.48, 21.27) | 18.78 (17.64 - 20.65) | 0.18 |
| CtOLS aspect ratio | 2.12 (2.04, 2.36) | 2.24 (2.05 - 2.37) | 0.24 |
| **Immunohistochemistry/TUNEL** | |  |  |
| FGF23/B.Ar (number of osteocytes/mm^2^) | 4.2 (0.3, 14.6) |  | |
| FGF23 (% tissue area) | 0.104 (0.073, 0.198) |  |  |
| FGF23 (staining intensity) | 0.805 (0.737, 0.866) |  |  |
| DMP1 (% tissue area) | 0.378 (0.269, 0.687) |  |  |
| DMP1 (staining intensity) | 0.866 (0.854, 0.902) |  |  |
| MEPE (% cells positive) | 6.9 (5.1, 9.4) |  |  |
| Sclerostin (% tissue area) | 0.084 (0.073, 0.166) |  |  |
| Sclerostin (staining intensity) | 0.868 (0.822, 0.928) |  |  |
| Bone TUNEL (% cells positive) | 6.5 (2.0, 14.6) |  |  |
| Marrow TUNEL (% cells positive) | 5.5 (1.8, 10.7) |  |  |

Data are reported as median (interquartile range).

*Note that the reference values for BMDD and most OLS are obtained on the same biopsy samples as the reference values for bone histomorphometry.

**Supplemental Table 3:**

1. **General Linear Model for Prediction of Trabecular (Tb) CaMean**

| **Variable** | **Parameter Estimate** | **Standard Error** | **P value** |
| --- | --- | --- | --- |
| **Intercept** | 21.40 | 0.44 | 0.001 |
| **BFR/BS** | -0.03 | 0.01 | <0.001 |
| **FGF23/B.Ar** | 0.08 | 0.04 | 0.04 |
| **Sclerostin/B.Ar** | 2.03 | 1.81 | 0.28 |
| **Bone TUNEL** | -0.02 | 0.03 | 0.58 |

1. **General Linear Model for Prediction of Trabecular (Tb) CaLow**

| **Variable** | **Parameter Estimate** | **Standard Error** | **P value** |
| --- | --- | --- | --- |
| **Intercept** | 11.08 | 3.09 | 0.003 |
| **BFR/BS** | 0.20 | 0.04 | <0.001 |
| **FGF23/B.Ar** | -0.62 | 0.25 | 0.024 |
| **Sclerostin/B.Ar** | -10.17 | 12.59 | 0.43 |
| **Bone TUNEL** | 0.12 | 0.23 | 0.60 |

**Supplemental Table 4.** Individual patient demographics. Samples were ordered by increasing rates of bone formation.

| **Age** | **Sex** | **Underlying kidney disease** | **FGF23/B.Ar** | **BFR/BS** | **TbCaMean** | **Cortical OLS.Ar** |
| --- | --- | --- | --- | --- | --- | --- |
| 16.9 | female | Unknown | 12.7 | 0.0 | 21.63 | 2.17 |
| 19.0 | female | Dysplasia | 17.9 | 0.0 | 20.91 | 2.36 |
| 25.0 | male | Dysplasia | 19.4 | 0.0 | 22.16 | 2.29 |
| 20.1 | male | Unknown | 0 | 0.3 | 22.48 | 1.98 |
| 20.9 | male | Cystinosis | 7.6 | 3.3 | 23.23 | 2.06 |
| 28.7 | male | Hypertension | 10.4 | 5.2 | 22.29 | 2.12 |
| 18.0 | male | ADPKD | 24.2 | 8.0 | 21.02 | 2.96 |
| 17.0 | male | Bilateral cystic dysplasia | 1.4 | 9.6 | 21.37 | 2.55 |
| 24.0 | female | Dysplasia | 30.7 | 16.7 | 22.48 | 2.04 |
| 15.7 | female | Unknown | 2.7 | 21.8 | 21.90 | 2.38 |
| 16.5 | male | Neurogenic bladder | 1.0 | 26.8 | 20.41 | 2.12 |
| 14.4 | male | Unknown | 0.5 | 31.3 | 20.35 | 2.05 |
| 13.8 | male | Posterior urethral valve | 0 | 31.5 | 18.94 |  |
| 8.6 | female | Obstructive uropathy | 16.8 | 31.9 | 21.32 | 2.40 |
| 20.4 | male | Alport's syndrome | 0.3 | 63.2 | 20.64 | 2.06 |
| 17.8 | female | MPGN II | 11.2 | 63.9 | 22.31 | 2.52 |
| 18.3 | male | Alport's syndrome | 0.3 | 67.7 | 18.75 | 1.94 |
| 16.4 | male | Bilateral renal dysplasia | 0.1 | 68.7 | 20.09 | 1.82 |
| 15.4 | male | FSGS | 12.4 | 74.0 | 20.64 | 2.10 |
| 10.5 | male | FSGS | 1.1 | 77.3 | 19.88 | 1.90 |
| 14.4 | female | FSGS | 0.1 | 79.8 | 19.58 | 2.04 |
| 18.9 | male | Posterior urethral valve | 16.4 | 92.1 | 20.46 | 2.26 |
| 15.0 | male | Immune complex glomerulonephritis | 5.6 | 92.2 | 18.99 | 2.22 |
| 19.3 | male | FSGS | 0.3 | 110.8 | 18.51 | 2.00 |
