# Supplementary figures and images for: Impaired bone matrix maturation and mineralization are prevalent in adolescent end-stage kidney disease
Source: JBMR Plus. 2026 Mar 12;10(5):ziag036. doi: 10.1093/jbmrpl/ziag036 (PMC13050508; doi:10.1093/jbmrpl/ziag036)

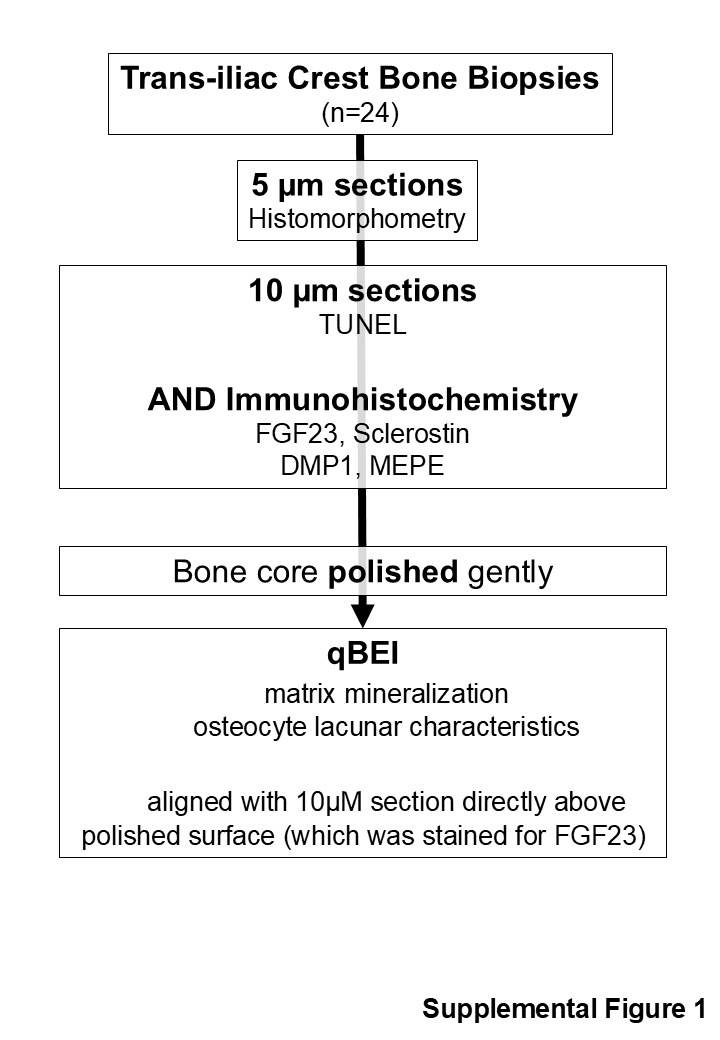

Supplement: fig9new_ziag036 [file fig9new_ziag036.jpeg]

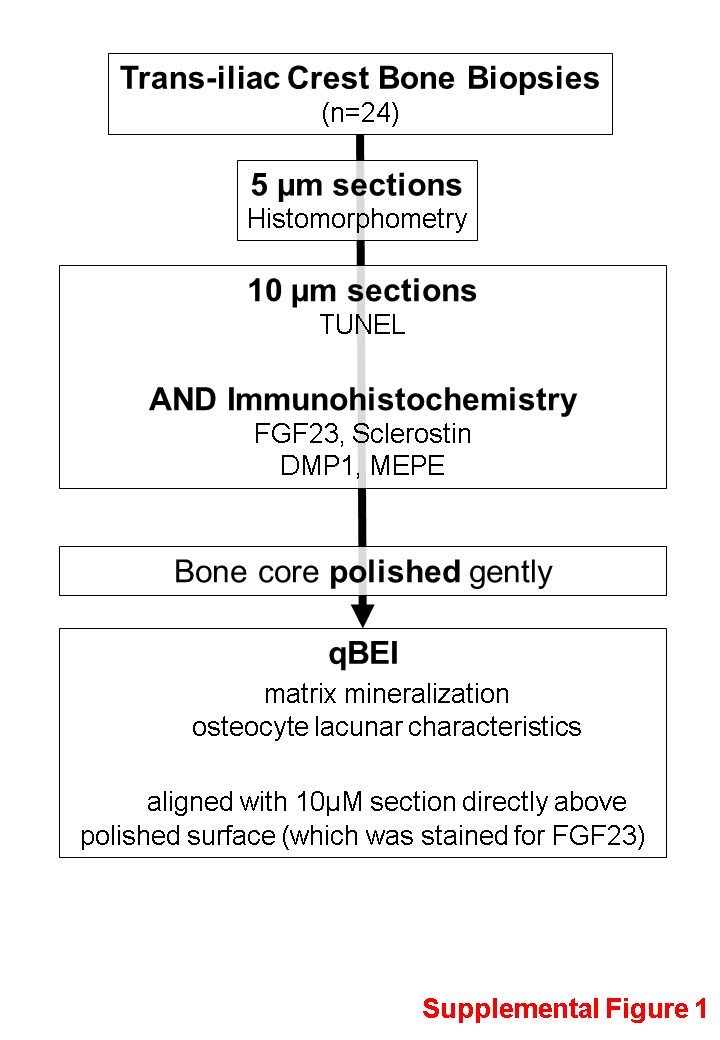

Supplement: Slide9_ziag036 [file slide9_ziag036.jpeg]
